# A single patient reported outcome measure for acquired brain injury, multiple sclerosis & Parkinson’s disease

**DOI:** 10.1371/journal.pone.0251484

**Published:** 2021-06-04

**Authors:** Ben Carter, Chloe Hayes, Alexander Smith, Anna Pennington, Michelle Price, Owen Pearson, Silia Vitoratou, Jonathan Hewitt

**Affiliations:** 1 Department of Biostatistics and Health Informatics, Institute of Psychiatry, Psychology & Neuroscience, King’s College London, London, United Kingdom; 2 School of Sport, Exercise and Health Sciences, Loughborough University, Leicestershire, United Kingdom; 3 Division of Population Medicine, School of Medicine, Cardiff University, Cardiff, United Kingdom; 4 Aneurin Bevan University Health Board, South Wales, United Kingdom; 5 Powys Teaching Health Board, Mid Wales, United Kingdom; 6 Swansea Bay University Health Board, Port Talbot, United Kingdom; University of Bologna, ITALY

## Abstract

**Objective:**

To determine psychometric properties of the PROMIS-10 and Standard Stroke Question Set (by International Consortium for Health Outcome Measures) presented as a new 15-item Patient Related Outcome (PRO), for patients with: acquired Brain Injury (ABI), Multiple sclerosis (MS) and Parkinson’s disease (PD).

**Methods:**

In an eight centre, UK wide, cross-sectional study we approached patients during their routine follow-up to complete: a disease-specific instrument (European Brain Injury Questionnaire, Multiple Sclerosis Impact Scale, and Parkinson’s disease questionnaire); General Health questionnaire with a Quality of life measure (EQ-5D); and PRO. We validated the PRO using factor analysis to define the latent construct domains, then calculated the internal consistency (Cronbach’s-α), and construct validity (correlation).

**Results:**

There were 340 patients with ABI (N = 91, median age = 55.1, 41% female), MS (N = 99, age = 58.9, 69%) and PD (N = 150, age = 74.5, 40%). Factor analysis suggested the PRO offered three domains of: physical health; functionality-capacity and mental health. All factors correlated strongly with the three disease-specific instruments, and the overall PRO had a large correlation with the EQ-5D (correlation>0.8) offering good construct validity and excellent internal consistency (∝>0.89).

**Interpretation:**

The PRO offered promising psychometric properties and could be used in place of disease specific questionnaires for patients with ABI, MS, and PD. The PRO has three construct domains, describing patients’: mental health; physical health; and functional-capacity, and may be used in routine clinical practice. The PRO offered both relevance to each of the three separate neurological conditions and generalisability across all the conditions, increasing its utility.

## Introduction

It is vital to place the patient at the centre of healthcare investigation, and a wide range of patient reported outcome measures (PROMs) have been developed to capture patients’ own perception of their health and quantify outcomes that are relevant to them [1–4]. PROMs may be disease-specific or generic [5–9]. While disease-specific PROMs are useful in assessing the specific symptoms of disease, these tend to be lengthy, difficult to complete and result in a poor response rate [1]. Each disease specific PROM also requires dedicated training and familiarisation. Conversely, being disease specific, they cannot be used or compared across different conditions [10–12]. Other advantages in using a Generic PROM tend to be shorter, they may be used across a range of conditions, require less training, and offer utility across different diseases, for example the EQ-5D [13]. However, these measures are often seen as less credible than disease-specific measures, with lower face validity [5].

In PD, ABI and MS, there are a range of disease specific PROs [14–16], They range in length, complexity and the amount of validation which they have undergone. These three common chronic neurological conditions, while different, do present with a large degree of overlapping symptoms, for example, reduced mobility, impaired continence and impairment of higher-level cognitive function. Therefore, the potential exists for creating an easy to use but transferable PRO which is shorter, easier to complete.

The Patient-Reported Outcomes Measurement Information System 10-Question (PROMIS-10) has utility and has been validated with different neurological conditions (stroke, Parkinson’s disease). However, the PROMIS-10 is rarely used in outpatient neurology clinics, or within neurological research, where physician engagement seen as a key challenge [17–19].

To improve patient reported outcomes in Stoke, the International IHCOM [20] undertook a Delphi exercise using a mix of patients, caregivers and stroke clinicians to develop a new 15-item Patient Related Outcome (herein called a PRO) [3]. This consisted of the established and validated PROMIS-10 with five additional items relevant to stroke survivors. This PRO was found to be feasible for patients to complete and helpful to their clinicians. It was successfully used in a UK wide study of over 2000 stroke survivors, but it has not previously been psychometrically tested within a stroke sample [18].

The aims of this study were to determine whether this PRO was relevant to patients, scientifically valid, easy to use and could provide a consistent outcome measure on which to assess improvements (or otherwise) across three common neurological conditions: Parkinson’s disease (PD); Multiple Sclerosis (MS); and Acquired Brain Injury (ABI). The objective of this study was to validate this PRO in patients with these common neurological conditions, through psychometric analysis to establish its factor structure, reliability and validity.

## Methods

This was a cross-sectional study of three neurological cohorts carried out in eight sites in England and Wales from August 2017 to January 2018. The neurological conditions research study was approved by Health Care Research wales NHS research ethics committee on 15^th^ March 2017 (Research Ethics Committee number 17/WA/0023).

The study inclusion criteria included anyone aged over 18 years of age with a diagnosis of ABI, PD or MS, for 1 year or longer.

### Instruments and procedures

All potentially eligible participants were selected from each local site and approached for entry into the study by their responsible physician. Across all sites potentially eligible participants were contacted via post to provide written informed consent. Each patient was asked to complete a demographic information, PRO, a disease specific questionnaire and the generic EuroQol-5D questionnaire [6]. The three disease specific PROMs were; the European Brain Injury Questionnaire [19] completed by those with an Acquired Brain Injury; the Multiple Sclerosis Impact Scale v2 [21] completed by those with Multiple Sclerosis; and the Parkinson’s disease questionnaire [22] completed by those with Parkinson’s disease.

### Patient Related Outcomes for neurological diseases (PRO)

The Patient Related Outcomes for Neurological Diseases (PRO) is a composite measure derived by the Stroke community from three measures: the Patient-Reported Outcomes Measurement Information System 10-Question (PROMIS-10 Short Form; [23]), the RiksStroke questionnaire [24] and the International Consortium for Health Outcomes Measurement (ICHOM; [20]).

#### PROMIS-10 global health

The PROMIS-10 is a validated self-reported ten item questionnaire. Each item is scored on a 5-point ordinal scale, with 1 indicating poorer health. The scale has two domains of mental health and physical health, as well as a total composite score. The raw domain scores are converted to a T-score, normed to the general population in the U.S. (mean = 50, standard deviation = 10), such that a person with a score of 60 is one standard deviation healthier than the average [25].

#### RiKsStoke and ICHOM stroke set questionnaires

The RiksStroke Questionnaire consists of three questions (one three-point scale, and two binary items) related to walking, toileting, and being able to dress, and the International Consortium for Health Outcome Measures (ICHOM) Stroke Specific Question Set consists of two binary questions: having a feeding tube; and or communication problems.

It is these 15 items that comprise the PRO and are to be validated with three samples.

### Disease specific instruments

#### European Brain Injury Questionnaire

The European Brain Injury Questionnaire (EBIQ) is a validated self-reported 63-item questionnaire, measuring a range of difficulties experienced by brain-injured patients. Each item is scored on a three-point scale, indicating how often the patient has experienced a particular difficulty in the past month. The scale consists of eight subscales (somatic, cognitive, motivation, impulse, depression, isolation, physical, and communication), and a composite score. Missing item data were not scored within the subscales.

#### Multiple sclerosis impact scale v2

The Multiple Sclerosis Impact Scale v2 (MSIS-29) is a validated self-reported 29-item questionnaire measuring the physical and psychological impact of MS on patients’ daily lives. Each item is measured on a four-point ordinal scale, indicating how much the patient has been bothered by each symptom in the past 2 weeks. Scores for both domains are standardised to the common range, 0 to 100, with higher scores indicating higher levels of impact. Missing item data resulted in subscales being reported as missing.

#### Parkinson’s disease questionnaire

The Parkinson’s disease questionnaire (PDQ-39) is a validated, self-reported 39-item questionnaire measuring the difficulties in daily living experienced by patients with Parkinson’s disease. Items are measured on a 5-point ordinal scale, indicating how often the patient has experienced difficulties in the last month, across eight dimensions: Mobility; Activities of daily living; Emotional wellbeing; Stigma; Social Support; Cognitive impairment; Communications; and Bodily discomfort. Missing item data resulted in subscales being reported as missing.

### General health quality of life instrument

#### The EuroQol-5D

The EuroQol-5D (EQ-5D) is a validated generic self-reported QoL questionnaire, it has two parts. Part 1 consists of five domains (mobility, self-care, usual activities, pain/discomfort, anxiety/depression), which are rated on four-point scale. The digits for the 5 dimensions are combined to provide a 5-digit description of current health state. The composite EQ-5D health state scores were converted to a crosswalk index score using the EQ-5D index value calculator (version 1.1; [13]). The crosswalk index can be interpreted such that 1 reflects a ‘perfect’ health score. The second part asks the respondent to rate their health on a visual analogue scale (VAS) from 0 to 100.

## Data analysis

The cohort demographics were described and reported consistent with the STROBE checklist [26]. Missing item level data were dealt with consistent to each specific validated instrument. A frequency distribution of the PRO responses was completed for each cohort. Non-parametric tests were used to assess gender differences within the PRO.

### Factor analysis

The endorsement of responses to each item was checked for differentiation, to identify potential problematic items. To describe the most efficient classification of the latent domains and model structure, item factor analysis (factor analysis for categorical data), due to mixed type categorical data, was undertaken on the PRO, using robust weighted least square (WLSMV) estimator [27]. An exploratory factor analysis (EFA) was carried out on the largest cohort (PD), following the 10:1 response to item ratio that is suggested by the literature [28]. Confirmatory factor analysis was used to test the model structure that emerged from EFA using the remaining two cohorts (ABI and MS). For CFA, the minimum sample size has been suggested to be a 5:1 response to item ratio, with a suggestion of *N*>100 [29], however the ideal is 10:1. Measures of absolute and relative fit were used to evaluate the overall model fit across the three cohorts. These measures were the relative chi-square (χ^2^/df: values close to 2 indicate close fit; [30]), the Root Mean Square Error of Approximation (RMSEA, values less than 0.08 are required for adequate fit, values of less than 0.05 indicate close fit; [31]), the Standardized Root Mean Residual (SRMR, values below 0.08 indicate adequate fit and values below 0.05 suggest close fit; [32]) the Tucker-Lewis Index (TLI, values higher than 0.9 are required for adequate fit, and above 0.95 for close fit; [33]) and the Comparative Fit Index (CFI, values higher than 0.9 are required for adequate fit and above 0.95 are required for close fit; [34]). Mplus software [35] was used in all factor analyses.

### Internal consistency and validity

The internal consistency of the final PRO was evaluated by Cronbach’s alpha coefficient (α; [36]), the value of alpha if item deleted (AID), and the item total correlation values (ITC) To offer adequate reliability alpha coefficients were required to be greater than 0.7, ITC greater than 0.3 but not higher than 0.8; [37]), and AID lower than Cronbach’s alpha value. McDonald’s omega (ω) will be evaluated, due to the mixed type categorical data in the PRO, the same criteria is followed as alpha [36]. Convergent validity was measured by correlating the PRO domains with the subdomains of the disease specific instruments, as well as with the index score and the VAS of the EQ-5D. The correlations were interpreted as small <0.2, moderate <0.5 and large of <0.7 [38]. Higher significant correlations values indicate greater evidence for validity, with large effect providing strongest evidence. The statistical software SPSS version 25.0 was used [39].

## Results

### Sample characteristics

A total of 429 participants consented to the study, and of those 85% (150/177), 71% (91/128) and 80% (99/124) returned questionnaires (PD, ABI and MS respectively). Of those that responded, 40% of the PD cohort were female (60/150), 41% of the ABI population were female (37/91) and 69% of the MS population were female (68/99) and 23% of those with PD (34/150), 30% of those with ABI (27/91) and 12% of those with MS(12/99) required care assistance. The median age (Q1, Q3) of the PD cohort was 74.5 (68.0, 79.3) years old, with a range of 42 to 100 years old, ABI cohort was 55.1 with a range from 21 to 97 (43.0, 63.6) and median age of the MS cohort was 59.0 (48.9, 68.4), ranging from 26 to 86 years old. The median EQ-5D health utility for the three cohorts was found to offer typical disease severity 0.59, 0.63 and0.52, for PD, ABI and MS (S1 Table). The cohort appeared to be typical and representative of the wider population of patients from the three neurological diseases. Incomplete responses were omitted from the dataset, as complete responses were required for factor analysis, for PD cohort 15 responses were removed, for ABI 18 responses were removed and for the MS cohort 10 responses were removed. This led to a sample for psychometric analysis of 135/150 participants of the PD cohort, 73/91 of the ABI cohort and 89/99 of the MS cohort.

### PRO response

The participant response per cohort, of the PRO are shown in (S2A–S2C Table). Within the ABI and MS cohorts, levels of general health, physical health, mental health, quality of life, social life and social activities were mostly rated as fair, and for the PD cohort these aspects were typically rated as good. Of those participants with PD, 40% felt they were able to either completely or mostly carry out physical activities. Similarly, of the individuals with ABI, 42% completely or mostly able to carry out physical activities. Whereas, this was not the case for participants with MS, of which 15% felt these activities could be carried out completely or mostly. Emotional problems were common (PD 39%, ABI 40% and MS 40%). Most respondents reported moderate levels of fatigue across the three cohorts (PD 57%, ABI 37% and MS 48%).

For the three RiksStroke questions, there were a range of responses. For example, in PD 30% needed help with dressing, in ABI, 18% needed help to go to the toilet and in MS, 47% either could not walk or needed help. S3 Table offered no evidence of differences for the PRO-by-gender (p>0.05). The participant responses appear typical of patients with these conditions.

### Psychometric statistical analysis

#### Exploratory Factor Analysis (EFA)

One item from the PRO (tube feeding) was omitted from the factor analysis due to having a poor discrimination since only one person answered ‘yes’ to having a feeding tube per cohort.

The PD cohort was used for EFA of the PRO, fitting up to 3 factors. The sample correlation matrix exhibited two eigenvalues above greater than one (8.103, 1.468; see S1 Fig), and thus up to two factors may be extracted [40]. Adequate fit was achieved at both the two- and three-factor solutions, with an improved fit achieved at the three-factor solution, according to the goodness of fit indices (Table 1).

**Table 1:** Goodness of fit indices for exploratory factor analysis and confirmatory factor analysis models across all cohorts.

Cohort	Model	rel χ^2^	RMSEA (p-close)	CFI	TLI	SRMR
PD (N = 135)	*EFA*: *one factor*	2.7	0.108	0.97	0.96	0.104
	*EFA*: *two factors*	1.9	0.080	0.98	0.98	0.065
	***EFA*: *three factors***	**1.6**	**0.062**	**0.99**	**0.99**	0.048
ABI (N = 73)	*CFA*: *one factor*	4.0	0.201 (<0.001)	0.89	0.87	0.192
	*CFA*: *two factors*	3.8	0.178 (<0.001)	0.89	0.87	0.171
	***CFA*: *three factors***	**2.9**	**0.162 (<0.001)**	**0.93**	**0.91**	**0.166**
MS (N = 89)	*CFA*: *one factor*	2.5	0.131 (<0.001)	0.94	0.93	0.116
	*CFA*: *two factors*	1.9	0.097 (0.001)	0.97	0.96	0.092
	***CFA*: *three factors***	**1.6**	**0.083 (0.031)**	**0.98**	**0.97**	**0.086**

*Note*. The two-factor solution did not provide an adequate model structure due to high cross loadings, as such the model was rejected.

#### Confirmatory factor analysis

The ABI and MS cohorts were used to confirm the EFA factor structure, the cohorts fell below the minimum sample and present as first evidence. The model fit for both cohorts was improved as the number of factors was increased from one to three. The one-factor solution did not provide adequate fit for either cohort. The 3-factor structure, with factor loadings shown in Table 2, provided adequate fit for the MS cohort, but the fit was more marginal for the ABI cohort.

**Table 2:** Standardised factor loadings for exploratory factor analysis of the PD sample and confirmatory factor analysis (within brackets ABI / MS) for the 3-factor solution.

Item	PH	FN	MH
G1—General health	0.74 (0.98 / 0.78)	**0.08**	**0.10**
G2—Quality of life	0.84 (0.79 / 0.95)	**-0.09**	**0.18**
G3—Physical health	0.82 (0.91 / 0.79)	**-0.01**	**0.18**
G8 –Fatigue	0.37 (0.68 / 0.60)	**0.30**	**0.21**
G6—Physical activities	**0.18**	0.60 (0.96 / 0.98)	**0.19**
G7 –Pain	**0.36**	0.37 (0.80 / 0.40)	**-0.04**
Rik1—Can you Walk unaided	**0.08**	0.86 (0.67 / 0.83)	**-0.16**
Rik2—Can you go to the toilet unaided	**-0.21**	0.95 (0.66 / 0.84)	**0.19**
Rik3—Can you dress unaided	**0.03**	0.86 (0.79 / 0.90)	**0.02**
G4—Mental health	**0.04**	**0.04**	0.76 (0.94 / 0.59)
G5—Social life	**0.14**	**-0.05**	0.74 (0.80 / 0.87)
G9—Social activities	**0.13**	**0.31**	0.60 (0.86 / 0.95)
G10—Emotional problems	**-0.02**	**-0.03**	0.78 (0.70 / 0.62)
ICHOM—Communication Problems	**-0.27**	**-0.09**	0.31 (0.56 / 0.75)

Note. PROMIS-10 items are coded by a G, RiksStroke and ICHOM items are identified by name. PH = Physical Health; FN = Functionality; MH = Mental Health. Factor loadings in bolded text are EFA loadings of items per factor.

#### Interpretation of the factor analysis

The three-factor solution contained three latent domains within PRO, physical, emotional, and functional capacity (Table 2). The first factor related to self-perceived levels of physical health (PH), containing four items from the PROMIS-10: quality of life, physical health, general health, and fatigue. The second factor grouped three RiksStroke items and two PROMIS-10 items, relating to daily functional capacity (FN; going to the toilet, dressing, walking, and general physical activities) and pain rating. These items make up a ‘functional capacity’ factor. The third factor explained mental health (MH) status, grouping psychosocial aspects of health (mental health, social life, social activities, emotional problems and communication). Item 15 (communication problems) may be problematic due to a low loading score of 0.31 [41]. Factor scores are the sum of each item within factor, and lower scores were indicative of a poorer level of health/functioning.

#### Reliability and validity

Using the latent domains identified from the factor analysis we estimated the reliability and validity of the latent factors (physical health, functional capacity and mental health) and composite score of the PRO. The internal consistency of the physical health (PH) factor was good for the ABI and MS cohorts (∝>0.80) and excellent for the PD cohort (∝ = 0.89). Cronbach’s alpha for the functional capacity (FN) factor was acceptable for the MS and PD cohorts (∝>0.70) but was less established in the ABI cohort (∝ = 0.61). The reliability of the mental health (MH) factor was good across all three cohorts (∝>0.79). Cronbach’s alpha coefficient was excellent for PRO total score across all three cohorts (∝≥0.89). Within factors PH and MH, alpha could not be improved by deletion of any item and the ITC varied between 0.34 and 0.89, and no items were identified as problematic. Within the functionality factor, the question relating to pain was identified as a potential problematic item in cohorts ABI and MS, with the ITC less than 0.3 and AID suggesting that fit may be improved with removal of the item, but this was not a concern within the PD cohort. McDonald’s omega (ω) found similar findings for the PH factor, ω>0.8 across all cohorts. For the ABI and MS cohorts, omega for the FN factor was acceptable (ω≥0.75), and omega for FN was good in the PD cohort. The MH factor was found to have good internal reliability across the three cohorts, according to omega (ω>0.8).

Table 3 shows the correlations between PRO scores and EQ-5D, EQ VAS and the three cohort specific scales. For correlation between PRO scores and the EQ-5D, the PH and MH factors were moderately to highly correlated with EQ-5D for all cohorts (rho≥0.58), and the FN factor was highly correlated with EQ-5D across all three cohorts (rho>0.8). The EQ5D-VAS was at least moderately correlated with the PH factor across the 3 cohorts (rho = 0.87, 0.76, 0.64 for ABI, MS, and PD, respectively).

**Table 3:** Construct validity (Spearman’s correlation) between the PRO and RiksStroke, ICHOM, EQ-5D, EQ5D-VAS, and disease specific scales for each cohort.

	Physical	Functional	Mental	
	Health	capacity	Health	PRO Total score
Acquired Brain Injury				
RIK	0.20	0.55**	0.13	0.34*
ICHOM	0.24	0.32*	0.38**	0.34*
EQ-5D	0.65**	0.83**	0.65**	0.77**
EQ VAS	0.87**	0.67**	0.67**	0.83**
ABI-somatic	-0.73**	-0.55**	-0.63**	-0.69**
ABI-cognitive	-0.65**	-0.70**	-0.68**	-0.74**
ABI-motivation	-0.69**	-0.69**	-0.71**	-0.76**
ABI-impulse	-0.57**	-0.41**	-0.67**	-0.61**
ABI-Depression	-0.65**	-0.55**	-0.70**	-0.69**
ABI-Isolation	-0.68**	-0.64**	-0.74**	-0.73**
ABI-Physical	-0.71**	-0.78**	-0.71**	-0.81**
ABI-communication	-0.54**	-0.62**	-0.62**	-0.65**
ABI-core	-0.73**	-0.71**	-0.77**	-0.81**
Multiple Sclerosis				
RIK	0.50**	0.86**	0.42**	0.70**
ICHOM	0.26*	0.19	0.47**	0.38**
EQ-5D	0.65**	0.84**	0.64**	0.83**
EQ VAS	0.76**	0.57**	0.61**	0.74**
MSIS-29 –Physical	-0.70**	-0.77**	-0.64**	-0.81**
MSIS-29—Psychological	-0.73**	-0.57**	-0.81**	-0.81**
Parkinson’s Disease				
RIK	0.50**	0.82**	0.38**	0.64**
ICHOM	0.31**	0.23*	0.45**	0.39**
EQ-5D	0.69**	0.83**	0.58**	0.79**
EQ VAS	0.64**	0.57**	0.55**	0.69**
PDQ-39 –Mobility	-0.66**	-0.86**	-0.55**	-0.78**
PDQ-39—Activities of daily living	-0.62**	-0.70**	-0.53**	-0.70**
PDQ-39—Emotional well being	-0.62**	-0.55**	-0.70**	-0.72**
PDQ-39 –Stigma	-0.37**	-0.21*	-0.40**	-0.40**
PDQ-39—Social Support	-0.26**	-0.21*	-0.22*	-0.25*
PDQ-39—Cognitive impairment	-0.40**	-0.48*	-0.55**	-0.56**
PDQ-39—Communications	-0.45**	-0.43**	-0.60**	-0.59**
PDQ-39—Bodily discomfort	-0.43**	-0.60**	-0.48**	-0.59**
Total	-0.71**	-0.76**	-0.70**	-0.84**

*Note*.

*p≤0.05

**p≤0.01

Across the cohorts, all three factors correlated with the equivalent disease specific subdomains. Physical health scores of the PRO were moderately correlated with the somatic and physical scores of the EBIQ, physical MSIS-29 score and PDQ-39 mobility score. Functionality was moderately correlated with physical EBIQ and MSIS-29 scores, and PDQ-39 scores of mobility and activities of daily living. Mental health score was most correlated with the isolation, cognition, motivation and depression subscales of the EBIQ, MSIS-29 psychological score, and PDQ-39 emotional wellbeing score, from moderate to high correlations. Factors PH and MH were found to be moderately correlated with the core domain score of the EBIQ. All factors were highly correlated with PDQ-39 total score.

## Discussion

### Key findings

This study enrolled 340 patients from eight hospital trusts in the UK to the three cohorts. We found that the PRO offered three factors, which represented the domains of physical health, mental health, and functional capacity, with adequate, to good fit in the MS and PD cohorts. Overall, the PRO offered good internal consistency, and moderate convergent validity.

#### Concurrency with other literature

Researchers have previously assessed the psychometric utility of disease-specific instruments to address the needs of patients from the three neurological conditions included here. However, it has often been reported that the instruments do not report patients concerns adequately [5]. We have shown that our PRO has good psychometric properties may be used for three neurological conditions.

In the original validation of the PROMIS-10 by Hays et al. [42] the authors suggested (and CFA confirmed) a two-factor structure for the 10 items. A similar structure was identified from the item factor analysis of the 14-item PRO. Here, we compare the two solutions. The new PH factor consists of the previously physical loaded items Global01 (general health), Global03 (physical health) and Global08 (fatigue). In addition to these, the item Global02 (quality of life) was loaded to PH. In Hays et al. [42] this item cross loaded to the PH and MH factors with almost identical loadings (0.45). In our sample the item clearly loads on PH, suggesting patients with these specific diseases interpret quality of life in terms of physical health. Within the PROMIS-10, Global06 (physical activities) and Global07r (pain) loaded to the factor relating physical health. However, with the addition of the three RiksStroke questions, these items load now to a more specific Functionality factor. Finally, with respect to the new MH factor of the PRO, it consists of the items Global04 (mental health), Global05 (social life), and Global10 (emotional problems), again, similar to the results shown by Hays et al. [42]. In addition, the item Global09 (social activities) cross loaded to both factors [42], whereas in our data it loads more closely to the MH factor. We found the ICHOM item relating to communication problems also loads to the new MH factor, prompts to the fact that the factor is developed away from simply measuring mental health, to also encompassing aspects of psychosocial functioning. This is consistent with current view that both physical, and mental, and social functionality are the driving needs for older people [17, 43].

The PRO expands on the previous two factors from the PROMIS-10, capturing an important patient identified, aspect of daily wellbeing that can be impacted by all of the three neurological conditions. The addition of the Functionality factor, alongside both the Physical Health and Mental Health factors, can provide a more complete view of patient reported outcomes. The Functional factor resembles a patient’s ability to conduct day to day functional tasks exhibiting independence whilst pain free. We found that patients’ independence, and specifically their functionality, is important, which is consistent with other research [17].

We found that the PRO had validity with the EQ-5D, and previous authors had also found that the EQ-5D was reliable compared to other disease-specific questionnaires [44]. However, it has also be argued that the EQ-5D had inadequate detail to be used as a routine clinical follow up instrument [9, 45]. In contrast to the EQ-5D the PRO used in this study provides far more detail regarding specific symptoms that affect these three conditions. For example, details such as communication difficulties and it provides three separate domains of patient reported health: mental, physical and functional health.

Consistent with Hunter et al. [4], we found that a single instrument has the potential to represent a wider variety of long-term conditions to reduce the burden on patients, and offers efficiency to the healthcare system, as a harmonised measure consistent for studies of three neurological conditions. Patient’s responses to the PRO were consistent for each of the three conditions, which mirrored other findings from Esbjörnsson, Skoglund and Sunnerhagen [46].

#### Strengths and weaknesses

This is the first evaluation of the psychometric properties of a patient relevant outcome measure for multiple neurological diseases in the same study. The study consented patients exhibiting a typical background of patients of the three conditions. A limitation of the current study is that sample sizes were relatively small, of the three cohorts, the ABI and MS cohorts did not reach the minimum suggested sample size of 100 and further evidence of support should be identified [29, 47].

#### Implications on future clinical practice and research

This study’s findings suggest that the PRO is likely to reduce the burden for patients completing their follow-up in routine clinical practice, as well being straightforward for clinicians to interpret. Using a single, transferable assessment across three different conditions also offers health care providers advantages. For example, it is simple to use, and efficient to train staff in the use of a single assessment, and it may be useful in assessing changes in healthcare outcomes following changes in service delivery and design.

Measuring PRO across time periods is a key part of their function and future research should include serial measurements of the PRO to ensure that it has a sensitivity to detect changes in disease progression as well as assess the test-retest reliability of the PRO. Future research should also include a larger number of patients.

While basic interpretation of PRO domain scores would suggest that lower scores indicate an increased severity of impact, it is unclear how domain scores relate to severity of the neurological conditions, or how they compare to the general population. Further research is needed to determine a standardised score to the general population, and clinically meaningful thresholds to aid the interpretation.

## Conclusions

The PRO was found to offer both reliable and valid psychometric properties as a tool to measure patients’ symptoms. The instrument has three constructs: mental health; physical health; and functional capacity (or independence). Whilst cautious should be taken due to the size of the three cohorts, this study demonstrated first evidence that the PRO can be used in place of the disease specific instruments in at least three different neurological conditions. By demonstrating that the PRO can be used in a range of conditions it will hasten healthcare providers in implementing PRO in three conditions and improve patient centred care.

## Supporting information

S1 Fig Scree plot of the eigenvalues from the sample correlation matrix of the PD cohort. (DOCX)

S1 Table Scores on disease specific dimensions and generic measurements. (DOCX)

S2 Table PRO results for the included participants. Table 2A: ABI Results, Table 2B: MS Results, Table 2C: PD Results.
(DOCX)

S3 Table Descriptive summaries of the three factors (Physical Health [PH], Functional Capacity [FN], and Mental Health [MH]). Groups were compared using a non-parametric Mann Whitney U-test.
(DOCX)
