# Supplementary material for: A single patient reported outcome measure for acquired brain injury, multiple sclerosis & Parkinson’s disease
Source: PLoS One. 2021 Jun 4;16(6):e0251484. doi: 10.1371/journal.pone.0251484 (PMC8177510; doi:10.1371/journal.pone.0251484)
Supplement: S1 Fig — (DOCX) [file pone.0251484.s001.docx]

S1 Fig. Scree plot of the eigenvalues from the sample correlation matrix of the PD cohort.
