# Supplementary material for: A single patient reported outcome measure for acquired brain injury, multiple sclerosis & Parkinson’s disease
Source: PLoS One. 2021 Jun 4;16(6):e0251484. doi: 10.1371/journal.pone.0251484 (PMC8177510; doi:10.1371/journal.pone.0251484)
Supplement: S1 Table — (DOCX) [file pone.0251484.s002.docx]

| S1 Table: Scores on disease specific dimensions and generic measurements. | | | | |
| --- | --- | --- | --- | --- |
| Scale | Subscale | ABI  Median  (Q1, Q3) | MS  Median  (Q1, Q3) | PD  Median  (Q1, Q3) |
| PROMIS-10 | Physical Health | 11 (9, 15) | 10 (9, 12) | 12 (9,14) |
|  | Mental Health | 10 (7.25. 13) | 11 (8, 13) | 11 (9, 13) |
|  | Physical Health (t-score) | 37.4 (32.4, 47.7) | 34.9 (32.4, 39.8) | 39.8 (32.4, 44.9) |
|  | Mental Health (t-score) | 38.8 (32.6, 45.8) | 41.1 (33.8, 45.8) | 41.1 (36.3, 45.8) |
|  |  |  |  |  |
| PRO | Physical Health | 11 (8, 12) | 10 (7, 12) | 11 (8, 13) |
|  | Functionality | 13 (11, 15) | 11 (9,13) | 13 (10.5, 15) |
|  | Mental Health | 11 (9, 14) | 12 (10, 14.5) | 13 (11, 16) |
|  |  |  |  |  |
| EQ-5D-5L |  | 0.626  (0.341, 0.767) | 0.516  (0.211, 0.679) | 0.592  (0.408, 0.709) |
| EQ VAS |  | 65 (50, 80) | 60 (40, 75) | 65 (50, 80) |
|  |  |  |  |  |
| EQIB | Somatic | 2.00 (1.63, 2.50) |  |  |
|  | Cognitive | 2.08 (1.54, 2.46) |  |  |
|  | Motivation | 1.80 (1.40, 2.40) |  |  |
|  | Impulse | 1.77 (1.54, 2.15) |  |  |
|  | Depression | 1.78 (1.22, 2.11) |  |  |
|  | Isolation | 1.75 (1.25, 2.25) |  |  |
|  | Physical health | 1.75 (1.33, 2.00) |  |  |
|  | Communication | 2.00 (1.50, 2.50) |  |  |
|  | Core | 1.87 (1.51, 2.18) |  |  |
|  |  |  |  |  |
| MSIS | Physical |  | 61.7 (40.0, 78.3) |  |
|  | Psychological |  | 44.4 (25.9, 63.0) |  |
|  |  |  |  |  |
| PDQ | Mobility |  |  | 50.0 (17.5, 80.0) |
|  | Activities of daily living |  |  | 37.4 (16.7, 62.5) |
|  | Emotional wellbeing |  |  | 29.2 (12.5, 46.9) |
|  | Stigma |  |  | 12.5 (0.0, 31.3) |
|  | Social Support |  |  | 8.3 (0.0, 25.0) |
|  | Cognitive impairment |  |  | 31.3 (18.8, 50.0) |
|  | Communications |  |  | 16.7 (0.0, 41.7) |
|  | Bodily discomfort |  |  | 41.7 (25.0, 66.7) |
|  | Total |  |  | 30.1 (17.8, 45.1) |
|  | | | | |
