# Supplementary material for: A single patient reported outcome measure for acquired brain injury, multiple sclerosis & Parkinson’s disease
Source: PLoS One. 2021 Jun 4;16(6):e0251484. doi: 10.1371/journal.pone.0251484 (PMC8177510; doi:10.1371/journal.pone.0251484)
Supplement: S2 Table — Table 2A: ABI Results, Table 2B: MS Results, Table 2C: PD Results. (DOCX) [file pone.0251484.s003.docx]

S2 Table – PRO results for the included participants. Table 2A: ABI Results, Table 2B: MS Results, Table 2C: PD Results

S2A Table: ABI Results.

| **ABI** |  |  |  |  |  |
| --- | --- | --- | --- | --- | --- |
|  | **Poor** | **Fair** | **Good** | **Very good** | **Excellent** |
| G1 - General health | 7 (10%) | 29 (40%) | 23 (32%) | 11 (15%) | 3 (4%) |
| G2 - Quality of life | 6 (8%) | 27 (37%) | 26 (36%) | 11 (15%) | 3 (4%) |
| G3 - Physical health | 18 (25%) | 22 (30%) | 22 (30%) | 8 (11%) | 3 (4%) |
| G4 - Mental health | 20 (27%) | 24 (33%) | 14 (19%) | 14 (19%) | 1 (1%) |
| G5 - Social life | 14 (19%) | 32 (44%) | 10 (14%) | 15 (21%) | 2 (3%) |
| G9 - Social activities | 14 (19%) | 23 (32%) | 21 (29%) | 11 (15%) | 4 (6%) |
| G6 - Physical activities | **Not at all** | **A little** | **Moderately** | **Mostly** | **Completely** |
|  | 12 (16%) | 15 (21%) | 15 (21%) | 12 (16%) | 19 (26%) |
| G10 - Emotional problems | **Always** | **Often** | **Sometime** | **Rarely** | **Never** |
|  | 10 (14%) | 17 (23%) | 29 (40%) | 12 (16%) | 5 (7%) |
| G8 - Fatigue | **Very severe** | **Severe** | **Moderate** | **Mild** | **None** |
|  | 5 (7%) | 19 (26%) | 27 (37%) | 19 (26%) | 3 (4%) |
| G7 - Pain | **10 worst pain imaginable** | **7-9** | **4-6** | **1 to 3** | **0 no pain** |
|  | 0 (0%) | 18 (25%) | 20 (27%) | 18 (25%) | 17 (23%) |
| Rik1 - Walk | **Unable to walk** | **Able to walk with help** | | **Able to walk without help** | |
|  | 6 (8%) | 11 (15%) | | 56 (77%) | |
| Rik2 - Toilet | **I need help to go to the toilet** | | **I can manage going to the toilet without assistance** | | |
|  | 13 (18%) | | 60 (82%) | | |
| Rik3 - Dressing | **I need help dressing/undressing** | | **I can manage dressing/undressing without help** | | |
|  | 18 (25%) | | 55 (75 %) | | |
|  | **Yes** | | **No** | | |
| ICHOM – Feeding tube | 1 (1%) | | 72 (99%) | | |
| ICHOM - Communication Problems | 27 (37%) | | 46 (63%) | | |

Note: items with a prefix of G, and Rik, are from the PROMIS-10 and RikStroke respectively.

S2B Table: MS Results. Note, items with a prefix of G, and Rik, are from the PROMIS-10 and RikStroke respectively.

| **MS** |  |  |  |  |  |
| --- | --- | --- | --- | --- | --- |
|  | **Poor** | **Fair** | **Good** | **Very good** | **Excellent** |
| G1 - General health | 21 (24%) | 24 (27%) | 29 (33%) | 14 (16%) | 1 (1%) |
| G2 - Quality of life | 18 (20%) | 22 (25%) | 30 (34%) | 15 (17%) | 4 (5%) |
| G3 - Physical health | 23 (26%) | 38 (43%) | 16 (18%) | 9 (10%) | 3 (3%) |
| G4 - Mental health | 8 (9%) | 30 (34%) | 27 (30%) | 16 (18%) | 8 (9%) |
| G5 - Social life | 22 (25%) | 31 (35%) | 20 (23%) | 11 (12%) | 5 (6%) |
| G9 - Social activities | 22 (25%) | 36 (40%) | 19 (21%) | 7 (8%) | 5 (6%) |
| G6 - Physical activities | **Not at all** | **A little** | **Moderately** | **Mostly** | **Completely** |
|  | 24 (27%) | 30 (34%) | 22 (25%) | 6 (7%) | 7 (8%) |
| G10 - Emotional problems | **Always** | **Often** | **Sometime** | **Rarely** | **Never** |
|  | 8 (9%) | 22 (25%) | 36 (40%) | 18 (20%) | 5 (6%) |
| G8 - Fatigue | **Very severe** | **Severe** | **Moderate** | **Mild** | **None** |
|  | 4 (5%) | 30 (34%) | 43 (48%) | 12 (14%) | 0 (0%) |
| G7 - Pain | **10 worst pain imaginable** | **7-9** | **4-6** | **1 to 3** | **0 no pain** |
|  | 5 (6%) | 22 (25%) | 27 (30%) | 23 (26%) | 12 (14%) |
| Rik1 - Walk | **Unable to walk** | **Able to walk with help** | | **Able to walk without help** | |
|  | 20 (23%) | 21 (24%) | | 48 (54%) | |
| Rik2 - Toilet | **I need help to go to the toilet** | | **I can manage going to the toilet without assistance** | | |
|  | 24 (27%) | | 65 (73%) | | |
| Rik3 - Dressing | **I need help dressing/undressing** | | **I can manage dressing/undressing without help** | | |
|  | 35 (39%) | | 54 (61%) | | |
|  | **Yes** | | **No** | | |
| ICHOM – Feeding tube | 0 (0%) | | 89 (100%) | | |
| ICHOM - Communication Problems | 10 (11%) | | 79 (89%) | | |

Note: items with a prefix of G, and Rik, are from the PROMIS-10 and RikStroke respectively.

S2C Table: PD Results. Note, items with a prefix of G, and Rik, are from the PROMIS-10 and RikStroke respectively.

| **PD** |  |  |  |  |  |
| --- | --- | --- | --- | --- | --- |
|  | **Poor** | **Fair** | **Good** | **Very good** | **Excellent** |
| G1 - General health | 21 (17%) | 35 (28%) | 48 (38%) | 21 (17%) | 1 (1%) |
| G2 - Quality of life | 12 (10%) | 38 (30%) | 49 (39%) | 23 (18%) | 4 (3%) |
| G3 - Physical health | 25 (20%) | 47 (37%) | 36 (29%) | 17 (14%) | 1 (1%) |
| G4 - Mental health | 8 (6%) | 34 (27%) | 47 (37%) | 27 (21%) | 10 (8%) |
| G5 - Social life | 11 (9%) | 40 (32%) | 42 (33%) | 24 (19%) | 9 (7%) |
| G9 - Social activities | 15 (12%) | 40 (32%) | 46 (37%) | 22 (18%) | 3 (2%) |
| G6 - Physical activities | **Not at all** | **A little** | **Moderately** | **Mostly** | **Completely** |
|  | 13 (10%) | 30 (24%) | 33 (26%) | 33 (26%) | 17 (14%) |
| G10 - Emotional problems | **Always** | **Often** | **Sometime** | **Rarely** | **Never** |
|  | 4 (3%) | 21 (17%) | 49 (39%) | 36 (29%) | 16 (13%) |
| G8 - Fatigue | **Very severe** | **Severe** | **Moderate** | **Mild** | **None** |
|  | 5 (4%) | 15 (12%) | 72 (57%) | 29 (23%) | 5 (4%) |
| G7 - Pain | **10 worst pain imaginable** | **7-9** | **4-6** | **1 to 3** | **0 no pain** |
|  | 1 (1%) | 28 (22%) | 40 (32%) | 43 (34%) | 14 (11%) |
| Rik1 - Walk | **Unable to walk** | **Able to walk with help** | | **Able to walk without help** | |
|  | 5 (4%) | 30 (24%) | | 91 (72%) | |
| Rik2 - Toilet | **I need help to go to the toilet** | | **I can manage going to the toilet without assistance** | | |
|  | 16 (13%) | | 110 (87%) | | |
| Rik3 - Dressing | **I need help dressing/undressing** | | **I can manage dressing/undressing without help** | | |
|  | 38 (30%) | | 88 (70 %) | | |
|  | **Yes** | | **No** | | |
| ICHOM – Feeding tube | 1 (1%) | | 125 (99%) | | |
| ICHOM - Communication Problems | 28 (22%) | | 98 (78%) | | |

Note: items with a prefix of G, and Rik, are from the PROMIS-10 and RikStroke respectively.
