# Supplementary material for: A single patient reported outcome measure for acquired brain injury, multiple sclerosis & Parkinson’s disease
Source: PLoS One. 2021 Jun 4;16(6):e0251484. doi: 10.1371/journal.pone.0251484 (PMC8177510; doi:10.1371/journal.pone.0251484)
Supplement: S3 Table — Groups were compared using a non-parametric Mann Whitney U-test. (DOCX) [file pone.0251484.s004.docx]

| S3 Table: Descriptive summaries of the three factors (Physical Health [PH], Functional Capacity [FN], and Mental Health [MH]). Groups were compared using a non-parametric Mann Whitney U-test | | | | | |  |
| --- | --- | --- | --- | --- | --- | --- |
| Cohort |  | Females  Median (Q1, Q3) | Males  Median (Q1, Q3) | p-value | Total sample  Median (Q1, Q3) | |
| ABI | **PH** | 10.5(7, 12) | 11 (8, 12) | 0.31 | 11 (8, 12) | |
|  | **FN** | 13 (11, 16) | 13 (10, 15) | 0.60 | 13 (11, 15) | |
|  | **MH** | 11 (8.75, 14.25) | 11 (9, 15) | 0.80 | 11 (9, 14.5) | |
|  | **Total** | 33 (27, 42) | 33 (29, 40) | 0.83 | 33 (29, 41) | |
| MS | **PH** | 10 (7, 12) | 10 (8, 11.5) | 0.98 | 10 (7, 12) | |
|  | **FN** | 11.5 (9, 14) | 11 (10, 12) | 0.48 | 11 (9, 13) | |
|  | **MH** | 11.5 (9.25, 14.75) | 13 (10.5, 14.5) | 0.31 | 12 (10, 14.5) | |
|  | **Total** | 33 (25, 39.75) | 32 (29.5, 37) | 0.71 | 33 (26.5, 39) | |
| PD | **PH** | 11 (8, 12) | 11 (8, 13) | 0.63 | 11 (8, 13) | |
|  | **FN** | 13 (11, 14) | 13 (10, 15) | 0.71 | 13 (10.5, 15) | |
|  | **MH** | 13 (10, 15) | 13 (11, 16) | 0.34 | 13 (11, 16) | |
|  | **Total** | 37 (31.25, 41.75) | 37 (31, 45) | 0.72 | 37 (31, 43) | |
|  | | | | | |  |
